# Effect of fluocinolone acetonide (0.1%) treatment in oral lichen planus patients on salivary lactoferrin levels and *Candida* colonization: a prospective study

**DOI:** 10.1186/s12903-022-02096-3

**Published:** 2022-03-04

**Authors:** Nuttapong Saengprasittichok, Jeerus Sucharitakul, Oranart Matangkasombut, Chanwit Prapinjumrune

**Affiliations:** 1 Department of Oral Medicine, Faculty of Dentistry, Chulalongkorn University, Bangkok, 10330 Thailand; 2 Phaholpolpayuhasena Hospital, Kanchanaburi, Thailand; 3 Department of Biochemistry and Research Unit in Integrative Immuno-Microbial Biochemistry and Bioresponsive Nanomaterials, Faculty of Dentistry, Chulalongkorn University, Bangkok, Thailand; 4 Department of Microbiology and Center of Excellence on Oral Microbiology and Immunology, Faculty of Dentistry, Chulalongkorn University, Bangkok, Thailand

**Keywords:** Lactoferrin, *Candida*, Oral lichen planus, Topical steroid

## Abstract

**Background:**

Although topical steroids are an effective treatment for oral lichen planus, they can have suppressive effects on oral immunity and predispose the patients to *Candida* overgrowth. Lactoferrin is a crucial local immunity protein in the oral cavity with important antimicrobial activity. The aim of this study was to prospectively investigate salivary lactoferrin secretion levels and *Candida* colonization in oral lichen planus patients treated with fluocinolone acetonide 0.1% in orabase.

**Methods:**

Saliva samples were collected from 15 oral lichen planus subjects who had never received topical steroid treatment prior to this study and 15 healthy volunteers to determine their salivary lactoferrin levels using an enzyme-linked immunosorbent assay and to investigate the presence of oral *Candida* species at baseline and 3 months after treatment with fluocinolone acetonide 0.1% in orabase. Statistical analysis was performed to compare lactoferrin secretion and *Candida* colonization levels between the groups using the Mann–Whitney *U* test for independent data or the Wilcoxon Signed-Rank test for paired data.

**Results:**

The salivary lactoferrin secretion level was not significantly different between the control group and oral lichen planus patients or between before and after treatment with fluocinolone acetonide 0.1% in orabase (*P* > 0.05). *Candida* was detected in 11 (73.33%) healthy volunteers, 8 (53.33%) oral lichen planus patients before treatment, and 9 (60%) oral lichen planus patients after treatment with fluocinolone acetonide 0.1% in orabase. There was no significant difference in *Candida* counts between the groups (*P* > 0.05).

**Conclusion:**

Our study indicates that using fluocinolone acetonide 0.1% in orabase to treat oral lichen planus for 3 months did not affect salivary lactoferrin protein secretion or *Candida* carriage.

*Trial registration* The trial was registered at the Thai Clinical Trials Registry (TCTR identifier: TCTR20200723002).

## Background

Oral lichen planus (OLP) is a chronic inflammatory disease of the oral mucosa that results from an immunological disturbance [1, 2]. OLP lesions usually present on the oral mucosa with a variety of patterns, i.e. reticular, papular, plaque, atrophic, erosive, or bullous [3]. A burning sensation or pain when eating hot or spicy food, which affects their quality of life, is a common patient complaint. Due to advances in research and therapeutic methods, topical steroids, including clobetasol propionate, fluocinonide, and fluocinolone acetonide, are currently-used effective drugs for palliatively treating OLP [4]. However, using a topical steroid to treat OLP is a predisposing factor for increasing the incidence of an oral *Candida* infection. Several studies have reported the increased incidence of *Candida* infection in the oral cavity of OLP patients undergoing topical steroid treatment [5–8].

Lactoferrin (LF), also known as lactotransferrin, is an 80-kDa iron-binding glycoprotein of the transferrin family [9]. Lactoferrin is a component of various secreted fluids, such as milk, tears, saliva, bile, urine, and sweat, with a diverse range of biological activities [10]. This multifunctional protein is an effective host defense molecule. During the past decade, numerous studies have documented the important antimicrobial activities of lactoferrin in inhibiting bacterial growth, viral infection, and fungal infection [11–14]. In the oral cavity, lactoferrin is an important local innate immunity protein in the host defense system. A study on the secretion of antimicrobial proteins in the saliva of patients with oral candidiasis demonstrated that a significant decrease in lactoferrin correlated with the growth of *Candida* in the oral cavity [15].

*Candida* is a commensal microbe that is found in the oral cavity of healthy individuals [16]. However, this commensal microorganism can be an opportunistic pathogen when the host defense system is impaired. The antifungal mechanism of action of lactoferrin has been widely studied. The ability of lactoferrin to bind and sequester iron has been suggested to play an important role in its antifungal action in inhibiting *Candida albicans* and *Aspergillus fumigatus* growth [17, 18]. In addition, the iron-independent action of lactoferrin is also crucial for its antifungal effect. The direct interaction of this protein with the fungal cell wall surface, especially of *Candida* species, results in cell surface alterations and cell membrane permeability, inducing an apoptosis-like process [19–23].

The opportunistic infection by *Candida* species is a challenge when treating OLP with a topical steroid. Oral *Candida* infection exacerbates the clinical signs of OLP, and causes uncomfortable symptoms in the patients [24]. Although there are numerous reports concerning the antimicrobial effect of lactoferrin, little is known about the salivary levels of lactoferrin in relationship to the prevalence of oral *Candida* infection in OLP patients being treated with a topical steroid. Clarifying this relationship will provide new knowledge for preventing the development of oral candidiasis in patients undergoing topical steroid treatment. Thus, the aim of this study was to prospectively determine salivary lactoferrin levels and *Candida* colonization in OLP patients being treated with fluocinolone acetonide 0.1% in orabase (0.1% FAO) and to evaluate the relationship between *Candida* colonization and salivary lactoferrin levels.

## Methods

### Study population

In this observational study, the participants were recruited from patients at the Dental Hospital, Faculty of Dentistry, Chulalongkorn University, Bangkok, Thailand during October 2017 to October 2018. The sample size was calculated using a formula to estimate the sample size for comparing the difference between two dependent means for matched pairs (*n *_*p*_ = [((Z_α_ + Z_β_) σ_*d*_)/ES]^2^) [25]. The calculation based on the levels of lactoferrin before and after treatment in a pilot study, at α = 0.05 and β = 0.1, indicated that 15 participants per group were required. Fifteen patients with clinical and histopathological features of OLP were enrolled in the study. The diagnostic criteria for OLP was modified based on the clinical and histopathological definitions of van der Meij et al. and the American Academy of Oral and Maxillofacial Pathology, 2016 [26, 27] (Table 1). The inclusion criterion was OLP patients who had never used a topical steroid before participating in this study. The participants’ oral lesions were classified according to their clinical presentation as atrophic type or erosive type and were treated with 0.1% FAO applied 3 times/day for 3 months during the investigation. The control group consisted of 15 healthy volunteers. The exclusion criteria of both groups were signs of oral candidiasis at the beginning of the study, history of any disease or medical condition predisposing them to oral candidiasis or the promotion of *Candida* carriage, history of taking antifungal agents, using an antiseptic mouthwash, or smoking for within 1 month prior to the study. The appointments were scheduled at similar times of the day and the participants were asked to refrain from eating, drinking, and performing oral hygiene for 2 h before the evaluation appointment. To prevent experimental bias, the investigators were blinded to the clinical and demographic data of the participants.

**Table 1:** Oral lichen planus diagnostic criteria^a^

*Clinical criteria*	
Multifocal symmetric distribution	
White and red lesions exhibiting one or more of the following forms	
reticular/popular, atrophic, erosive (ulcerative), plaque, bullous	
Lesions are not localized exclusively adjacent to and in contact with dental restoration	
Lesions are not localized exclusively to the sites of smokeless tobacco placement	
Lesion onset does not correlate with the start of a medication or the use of cinnamon containing products	
*Histopathological criteria*	
Presence of band-like or patchy zone of cellular infiltration consisting mainly of lymphocytes in the lamina propria confined to the epithelium-lamina propria interface	
Signs of basal cell liquefactive degeneration	
Absence of epithelial dysplasia	

^a^Diagnostic criteria of OLP used in this study modified from van der Meij et al. [26] and the American Academy of Oral and Maxillofacial Pathology, 2016 [27]

### Saliva collection

Unstimulated whole saliva was collected at least 2 h after eating by having the participants spit their saliva into a sterilized tube until 2 ml was obtained or 10 min had elapsed [28]. After calculating the salivary flow rate, 1 ml of the collected saliva was centrifuged in a refrigerated centrifuge at 16,000 rpm for 20 min at 4 °C and stored at − 80 °C until used in the lactoferrin assays. Saliva samples from the OLP patients were obtained again after 3 months of 0.1% FAO treatment as described above.

### *Candida* isolation

One hundred µl and 500 µl saliva samples were spread separately on 2 Sabouraud dextrose agar plates (Oxoid, United Kingdom). After incubating the agar plates at 37 °C for 48 h, the number of colonies on each agar plate was counted and the colony-forming units (CFU) were calculated as CFU/ml of the collected saliva. The colonies were transferred from the Sabouraud dextrose agar plate onto a chromogenic *Candida* agar plate (Oxoid, United Kingdom). The agar plates were incubated at 37 °C for 48 h, and the *Candida* species were determined by the colony color according to the manufacturer’s instructions.

### Lactoferrin assays

The collected saliva was used to determine the salivary lactoferrin levels using an enzyme-linked immunosorbent assay (ELISA) (Human Lactoferrin ELISA kit; MyBioSource Inc, USA) that was performed according to the manufacturer’s instructions. Briefly, 100 µl saliva samples were added to the wells and allowed to react with the antibody at 37 °C for 90 min. The liquid in each microwell was then removed and incubated with 100 µl biotinylated detection antibody at 37 °C for 1 h. After aspirating and washing the microwells, 100 µl horseradish peroxidase conjugate was added and incubated at 37 °C for 30 min. The microwells were aspirated and washed again and incubated with 90 µl substrate reagent at 37 °C for 30 min. Finally, 50 µl stop solution was added into each microwell.

The salivary lactoferrin level was immediately detected with a spectrophotometer at 450 nm. A standard curve was used to determine the salivary lactoferrin concentration, which was then converted into the salivary lactoferrin secretion rate.

### Statistical analysis

Statistical analysis was performed using the Statistical Package for the Social Science (SPSS) version 22.0 (IBM Corporation, Armonk, NY, USA). The differences in salivary flow rate, salivary lactoferrin level, and the geometric means of colony-forming units in logarithmic notation were analyzed using the Mann–Whitney *U* test for independent data or the Wilcoxon Signed-Rank test for paired data as required. Furthermore, the prevalence and species of oral *Candida* were also compared between the groups using the Pearson chi-square test or Fisher’s exact test for independent data, or the McNemar test for paired data as required. Pearson’s correlation coefficient was used to evaluate the correlation between the amount of salivary *Candida* and salivary lactoferrin levels. *P*-values less than 0.05 were considered statistically significant.

## Results

### Participant demographics

The 15 OLP patients enrolled in this study comprised 13 females (86.7%) and 2 males (13.3%). The mean age of the OLP patients was 48.07 ± 9.66 years (range 24–62 years). The healthy volunteer control group consisted of 15 females with a mean age of 54.2 ± 9.63 years (range 42–72 years). The majority of the participants were female (age range 41–59 years-old). Most of the OLP patients (93.3%) presented with erythematous/atrophic oral lesions. None of the patients had skin lesions. The characteristics of the participants are summarized in Table 2.

**Table 2:** Participants’ characteristic (*N* = 30)

Characteristics	OLP patients	Healthy subjects
*Sex N (%)*		
Male	2 (13.3)	0 (0)
Female	13 (86.7)	15 (100)
*Age (Mean ± SD)*	48.07 ± 9.66	54.2 ± 9.63
*Age range N (%)*		
≥ 60	3 (20)	3 (20)
41–59	7 (46.7)	11 (73.3)
≤ 40	5 (33.3)	1 (6.7)
*Type of OLP N (%)*		
Atrophic	14 (93.3)	–
Erosive	1 (6.7)	–

### Salivary flow rate

The mean salivary flow rate in the healthy volunteers, OLP patients before treatment with 0.1% FAO, and OLP patients after treatment with 0.1% FAO was 0.54 ± 0.39, 0.36 ± 0.23, and 0.36 ± 0.18 ml/min, respectively (Table 3). There was no significant difference in salivary flow rate between the healthy volunteers and OLP patients before treatment (*P* = 0.213). Moreover, the salivary flow rate in the OLP patients before and after topical 0.1% FAO treatment was not significantly different (*P* = 0.955).

**Table 3:** Salivary flow rate and lactoferrin secretion

	Salivary flow rate (ml/min)		Lactoferrin concentration (ng/ml)		Lactoferrin flow rate (ng/min)	
	Mean ± SD	*P* value	Mean ± SD	*P* value	Mean ± SD	*P* value
*Healthy subjects*	0.54 ± 0.39	0.213^a^	2.36 ± 1.05	0.575 ^a^	1.11 ± 0.64	0.443 ^a^
*OLP patients*						
Before Tx with 0.1% FAO	0.36 ± 0.23	0.955^b^	3.49 ± 2.52	0.100 ^b^	1.13 ± 0.99	0.233 ^b^
After Tx with 0.1% FAO	0.36 ± 0.18		2.10 ± 0.88		0.67 ± 0.28	

^a^Mann-Whitney U test

^b^Wilcoxon Signed-Rank test

### Salivary lactoferrin concentration and secretion rate

The lactoferrin concentration and salivary lactoferrin flow rate were calculated (Table 3). The mean salivary lactoferrin concentration in the healthy volunteers, OLP patients before topical 0.1% FAO treatment, and OLP patients after topical 0.1% FAO treatment was 2.36 ± 1.05, 3.49 ± 2.52, and 2.10 ± 0.88 ng/ml, respectively. Furthermore, the mean salivary lactoferrin flow rate of the healthy volunteers, OLP patients before topical 0.1% FAO treatment, and OLP patients after topical 0.1% FAO treatment was 1.11 ± 0.64, 1.13 ± 0.99, and 0.67 ± 0.28 ng/min, respectively. The lactoferrin concentration and salivary lactoferrin flow rate between the OLP patients before topical 0.1% FAO treatment and healthy volunteers were not significantly different (*P* = 0.575 and *P* = 0.443, respectively). Furthermore, there was no significant correlation between the lactoferrin concentration and salivary lactoferrin flow rate in the OLP patients between before and after topical 0.1% FAO treatment (*P* = 0.100 and *P* = 0.233, respectively).

### Oral *Candida* colonization

To investigate the presence and species of oral *Candida*, salivary cultures were performed to determine the number of colony-forming units and the results are presented in logarithmic notation (Table 4). The salivary culture was positive for *Candida* in 11 (73.3%) healthy volunteers, 8 (53.3%) OLP patients before topical 0.1% FAO treatment, and 9 (60%) OLP patients after topical 0.1% FAO treatment. The mean amount of *Candida* in the saliva of the healthy volunteers, OLP patients before topical 0.1% FAO treatment, and OLP patients after topical 0.1% FAO treatment was 1.29 ± 1.14, 1.18 ± 1.22, and 1.28 ± 1.00 log CFU/ml, respectively. There was no significant difference in the amount of salivary *Candida* between the OLP patients before topical 0.1% FAO treatment and the healthy volunteers (*P* = 0.734). Importantly, the statistical analysis demonstrated no significant difference between the amount of salivary *Candida* in the OLP patients before and after topical 0.1% FAO treatment (*P* = 0.553).

**Table 4:** Prevalence and the mean *Candida* count in the saliva of OLP patients and healthy subjects

	Prevalence of *Candida*		*Candida* count (log CFU/ml)	
	N (%)	*P* value	Mean ± SD	*P* value
*Healthy subjects*	11 (73.3)	0.225^a^	1.29 ± 1.14	0.734^c^
*OLP patients*				
Before Tx with 0.1% FAO	8 (53.3)	1.00^b^	1.18 ± 1.22	0.553^d^
After Tx with 0.1% FAO	9 (60)		1.28 ± 1.00	

^a^Fisher’s exact test

^b^McNemar test

^c^Mann-Whitney U test

^d^Wilcoxon Signed-Rank test

### The presence of oral *Candida* and salivary lactoferrin levels

**Fig. 1:**
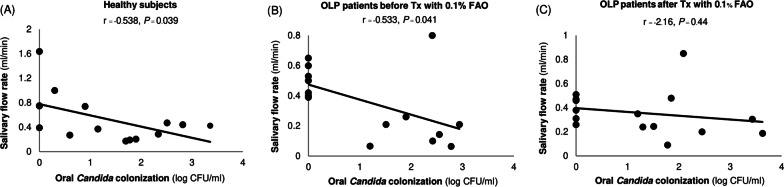
Correlation between the amount of salivary *Candida* and salivary flow rate in **A** healthy subjects, **B** OLP patients before treatment with 0.1% FAO, and **C** OLP patients after treatment with 0.1% FAO, analyzed by Pearson correlation coefficient. A *P* value of < 0.05 was considered statistically significant

**Fig. 2:**
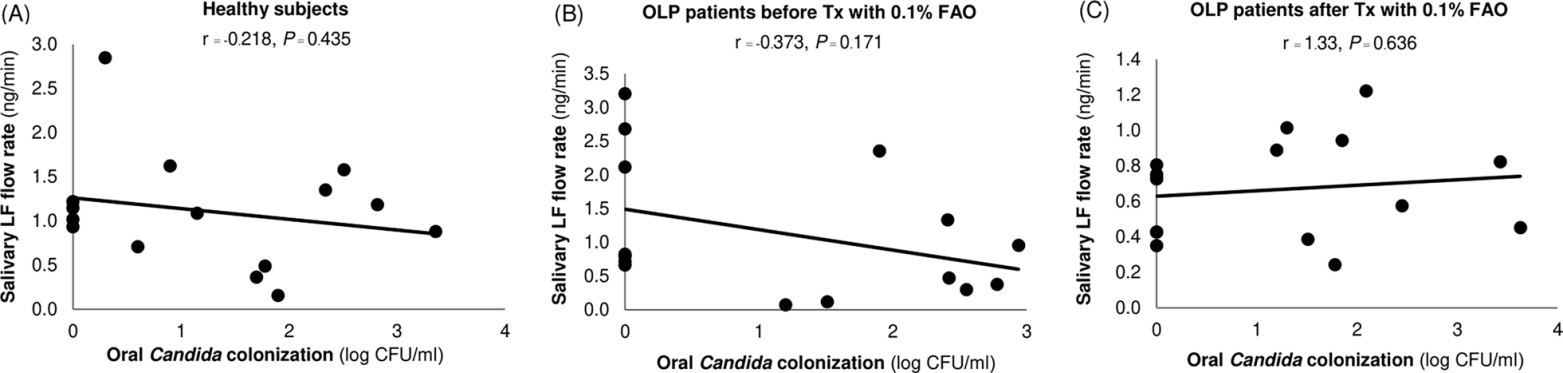
Correlation between the amount of salivary *Candida* and lactoferrin flow rate in **A** healthy subjects, **B** OLP patients before Treatment with 0.1% FAO, and **C** OLP patients after Treatment with 0.1% FAO, analyzed by Pearson correlation coefficient. A *P* value of < 0.05 was considered statistically significant

The association between the amount of salivary *Candida* and salivary flow rate or salivary lactoferrin levels was assessed using correlation analysis. The amount of oral *Candida* was negatively correlated with the salivary flow rate in the healthy volunteers and OLP patients before topical 0.1% FAO treatment (*P* = 0.039 and *P* = 0.041, respectively). However, there was no significant correlation in the OLP patients after topical 0.1% FAO treatment (*P* = 0.440) (Fig. 1) Furthermore, no relationship between the amount of oral *Candida* and salivary lactoferrin levels was observed in any group (*P* = 0.435, *P* = 0.171 and *P* = 0.636) (Fig. 2).

### *Candida* species in saliva

*Candida* species were identified from the saliva of the healthy volunteers and OLP patients (Table 5). Three *Candida* species were isolated. *Candida albicans* was the most common species detected in 8 (72.7%) healthy volunteers, 7 (87.5%) OLP patients before topical 0.1% FAO treatment, and 8 (88.9%) OLP patients after topical 0.1% FAO treatment. *Candida krusei* and *Candida glabrata*, non-albicans *Candida* species, were found in 3 (27.3%) healthy volunteers, 1 (12.5%) OLP patients before topical 0.1% FAO treatment, and 1 (11.1%) OLP patients after topical 0.1% FAO treatment. There was no significant difference in the prevalence of *Candida* species identified from the oral cavity of the healthy volunteers and OLP patients before topical 0.1% FAO treatment (*P* = 0.18). Moreover, the prevalence of *Candida* species identified from the oral cavity of the OLP patients before and after topical 0.1% FAO treatment was not significantly different (*P* = 0.931).

**Table 5:** *Candida* species in saliva of OLP patients and healthy subjects

*Candida* species	Healthy subjects No. (%)	OLP patients No. (%)		
		Before Tx with 0.1% FAO		After Tx with 0.1% FAO
*C. albicans*	8 (72.7)	7 (87.5)		8 (88.9)
*C. krusei*	3 (27.3)	0		0
*C. glabrata*	0	1 (12.5)		1 (11.1)
Total	11 (100)	8 (100)		9 (100)
*P* value^a^	0.18		0.931	

^a^Pearson chi-square test

## Discussion

Human saliva is a multifunctional secretion. This complex fluid plays important roles that influence oral health through its physical and chemical properties. Alterations in its quantity or quality directly affect the oral cavity. Quantitative changes in saliva have been described as a potential factor in the increased incidence of *Candida* infection in the oral cavity [29]. A decreased salivary flow rate reduces its flushing action to control of the amount of oral microorganisms. Impaired clearance of oral microbes results in the adhesion and colonization of oral microorganisms, including *Candida,* to the oral mucosa causing an increased risk of oral *Candida* infection. Consequently, increased colonization by oral *Candida* occurs with a high frequency in patients with xerostomia [30, 31]. Although our results demonstrated no significant difference in the salivary flow rate between the healthy subjects and OLP patients, a negative correlation between the salivary flow rate and the amount of salivary *Candida* in the healthy volunteers and OLP patients before topical 0.1% FAO treatment was observed in the present study.

Defending against the ingress of microorganisms into the body is one of the most important functions of human saliva. This secretion comprises a number of local immune proteins, such as lactoferrin, an antimicrobial component of saliva. The mechanism and biological role of lactoferrin have undergone numerous investigations in the past decade [11–14]. It has been demonstrated that the quantity of salivary lactoferrin protein is correlated with decreased saliva [28]. Our results indicated that neither salivary flow rate nor salivary lactoferrin levels were significantly decreased in the OLP patients in our study.

Topical steroids, such as 0.1% FAO, are widely recommended as the initial drug of choice for treating OLP. However, the most common adverse effect when treating OLP with a topical steroid is the increased adhesion and growth of *Candida* in the oral cavity [6]. Currently, the biological role of salivary lactoferrin in inhibiting *Candida* growth is unresolved. Decreased salivary lactoferrin may cause oral *Candida* overgrowth [15]. The relationship between decreased lactoferrin secretion and using a topical steroid was also demonstrated in an in vitro study using nasal and bronchial mucosa explants [32]. However, we found no correlation between using topical 0.1% FAO and a quantitative change in salivary lactoferrin in this study.

As previously mentioned, lactoferrin participates in the host defense system. Because lactoferrin is an iron-binding protein, its function requires the presence of iron in the saliva. A few studies have investigated the amount of iron in OLP patients’ saliva; however, the results have been inconsistent [33, 34]. Although a correlation between topical steroid use and iron level in oral fluid has not been determined, several studies have also demonstrated that lactoferrin can directly interact with the fungal cell wall in an iron-independent manner and this may play an important role in its antifungal activity against *Candida* [19–23]. Our study found no significant decrease in salivary lactoferrin levels nor increase in *Candida* colonization in the OLP patients who were undergoing topical 0.1% FAO therapy compared with healthy individuals. However, in cases of OLP treated with topical steroids that had an increased incidence of oral candidiasis, it is also possible that the infection may be due to the impairment of other factors.

*Candida* microbes living as normal flora in the oral cavity can be detected in approximately one-half of the world’s population and *Candida albicans* is the most frequently identified species in the oral cavity [6, 16]. Although none of the subjects had signs or symptoms of oral candidiasis in the present study, we observed the presence of oral *Candida* in over half of the subjects, and *C. albicans* was the most common species found in the oral cavity in both healthy participants and OLP patients, which is consistent with a previous study [6]. Interestingly, our study demonstrated a marked difference in the *Candida* species isolated from the saliva of healthy subjects and OLP patients. This finding should be evaluated in a future study.

The major strength of this study is the use of a prospective design. This allowed us to compare the levels of *Candida* colonization and salivary lactoferrin in OLP patients before and after a finite period of topical 0.1% FAO treatment in the same subjects. Moreover, we included only OLP patients who had never received any topical steroid therapy before the start of this study to minimize any effect from previous treatment. In addition, the OLP patients and healthy controls had no history of systemic conditions that are known to affect oral *Candida* colonization and lactoferrin levels. Although these strict inclusion and exclusion criteria limited the pool of the eligible sample population, these were an important strength of the present study.

Interestingly, although our results revealed a decreased lactoferrin concentration and lactoferrin flow rate in the OLP patients undergoing topical 0.1% FAO treatment compared with the other groups, there were no significant differences in salivary flow rate, lactoferrin concentration or salivary lactoferrin flow rate between the groups. However, these decreases did not result in a significant difference in the *Candida* count between these groups. Therefore, the limitations of the present study should be considered. Although the sample size in this study had sufficient statistical power according to the sample size calculation, the small sample size in each group may be a key limitation. Furthermore, the limited duration of the present study is also a concern. Thus, we suggest that these findings should be further investigated in future studies with a larger sample size and longer duration. The progress in further research will be helpful in our understanding the mechanisms and the biological role of salivary lactoferrin in inhibiting the occurrence of oral candidiasis in OLP patients undergoing topical steroid therapy. In the future, an advanced understanding of the pathogenesis of oral candidiasis may aid in developing new prevention methods that decrease the risk of oral candidiasis in patients undergoing topical steroid treatment.

## Conclusion

In summary, we found the normal secretion of salivary lactoferrin and no significant changes in oral *Candida* colonization in OLP patients after 3 months of 0.1% FAO treatment. Our results suggest that using 0.1% FAO in treating OLP may not result in a meaningful quantitative change in salivary lactoferrin levels and *Candida* carriage.

## Data Availability

All data generated or analysed during this study are included in this published article.

## Abbreviations

CFU: Colony-forming units
FAO: Fluocinolone acetonide in orabase
LF: Lactoferrin
OLP: Oral lichen planus
r: Correlation coefficient
SD: Standard deviation
Tx: Treatment
